# Forgotten No More—The Role of Right Ventricular Dysfunction in Heart Failure with Reduced Ejection Fraction: An Echocardiographic Perspective

**DOI:** 10.3390/diagnostics11030548

**Published:** 2021-03-19

**Authors:** Aura Vijiiac, Sebastian Onciul, Claudia Guzu, Alina Scarlatescu, Ioana Petre, Diana Zamfir, Roxana Onut, Silvia Deaconu, Maria Dorobantu

**Affiliations:** 1 Department of Cardiology, Carol Davila University of Medicine and Pharmacy, 050513 Bucharest, Romania; sebastian.onciul@gmail.com (S.O.); i_comanescu@yahoo.com (I.P.); silvia.iancovici@yahoo.com (S.D.); maria.dorobantu@gmail.com (M.D.); 2 Department of Cardiology, Emergency Clinical Hospital, 014461 Bucharest, Romania; claudia.paunescu91@gmail.com (C.G.); alina.scarlatescu@gmail.com (A.S.); diana_zam74@yahoo.com (D.Z.); onutroxana@yahoo.com (R.O.)

**Keywords:** right ventricle, heart failure with reduced ejection fraction, myocardial strain, three-dimensional echocardiography

## Abstract

During the last decade, studies have raised awareness of the crucial role that the right ventricle plays in various clinical settings, including diseases primarily linked to the left ventricle. The assessment of right ventricular performance with conventional echocardiography is challenging. Novel echocardiographic techniques improve the functional assessment of the right ventricle and they show good correlation with the gold standard represented by cardiac magnetic resonance. This review summarizes the traditional and innovative echocardiographic techniques used in the functional assessment of the right ventricle, focusing on the role of right ventricular dysfunction in heart failure with reduced ejection fraction and providing a perspective on recent evidence from literature.

## 1. Introduction

Heart failure (HF) remains a rising public health concern, with an estimated prevalence of almost 38 million individuals worldwide [1,2]. The total percentage of the population with HF is predicted to rise to 2.97% in 2030 [3]. Currently, HF is classified into HF with preserved, mid-range, or reduced ejection fraction (EF) [4], the latter being the most extensively studied.

Most of the previous research demonstrated the high prognostic value of left ventricular (LV) dysfunction [5], while the significance of right ventricular (RV) dysfunction in HF with reduced EF is less clear. This may be partly explained by the complex RV three-dimensional (3D) geometry, which makes its echocardiographic assessment challenging [6]; for this reason, the RV used to be called for quite a while “the forgotten chamber” [7]. However, during the last decade, RV dysfunction emerged as a strong predictor in HF and/or pulmonary hypertension [8,9], thus raising awareness of the importance of accurate assessment of RV performance.

In the era of multi-modality imaging, cardiac magnetic resonance (CMR) imaging remains the gold standard for RV quantification, despite the technical innovations in the field of echocardiography [10,11,12,13]. The prognostic role of CMR-derived RVEF in patients with dilated cardiomyopathy (DCM) and reduced LVEF is well established. In this population, RVEF was found to be an independent predictor of transplant-free survival [14], malignant arrhythmic events [15], cardiac death [16], and all-cause mortality [17].

However, the high cost and low availability of CMR hamper its unrestricted use on all patients with HF. By comparison, echocardiography is a bedside, widely available tool, and novel echocardiographic techniques such as myocardial strain imaging and three-dimensional (3D) echocardiography allow an accurate RV quantification that has been validated against (CMR) [18,19].

The aim of this review is to summarize the role of both conventional and novel echocardiographic parameters of RV function in patients with HF and reduced EF (HFrEF), while focusing on recent research.

## 2. The Echocardiographic Assessment of the Right Ventricle

The echocardiographic assessment of the RV faces several challenges: (1) the complex RV geometry; (2) its position behind the sternum; (3) the cumbersome endocardial tracing due to prominent trabeculations; and (4) its dependence on hemodynamic load and the RV–LV interdependence. In fact, some authors suggest that up to 20–40% of RV stroke volume results from the contraction of the LV [20]. The contraction pattern of the RV is sequential, starting at the inlet and progressing to the infundibulum [21]. The RV mechanics encompass a complex interplay between the longitudinal, radial, and antero-posterior shortening [22]. Traditionally, it was considered that the RV pump function was mainly driven by the longitudinal contraction. However, recent studies revealed that the radial and antero-posterior contractile components are equally important [23,24].

There is no ideal echocardiographic parameter for RV quantification [25], hence a thorough echocardiographic evaluation needs an integrative, multi-parametric approach from multiple acoustic windows, as suggested by current guidelines [26]. Conventional parameters assessing RV systolic function are tricuspid annular plane systolic excursion (TAPSE), tissue Doppler imaging (TDI)–derived tricuspid lateral annular systolic velocity (S’ wave), RV isovolumic acceleration, and RV fractional area change (FAC). The RV myocardial performance index (MPI) is a measure of global systolic and diastolic RV function. Innovative parameters for the assessment of the RV performance are derived from two-dimensional (2D) or 3D speckle-tracking echocardiography (STE) or 3D echocardiography: RV global and free wall strain and strain rate, as well as the 3D RVEF.

Most of these parameters (such as TAPSE, S’ wave, and RV strain) assess only the longitudinal RV function, while others (such as FAC) account for both the longitudinal and radial components of the RV contraction. However, most of the RV functional parameters neglect the contribution of the outflow tract contraction, potentially leading to an underestimation or overestimation of the global RV systolic performance [25]. This limitation is overcome by 3D RVEF, which integrates all the three components of RV mechanics, by reconstructing the RV endocardial surface independent of any geometric assumptions [27].

## 3. Tricuspid Annular Plane Systolic Excursion (TAPSE)

TAPSE is a highly reproducible, easy obtainable parameter [28] of RV longitudinal function, which is acquired by placing the M-mode line at the lateral tricuspid annulus in the apical four-chamber view. The vertical excursion of the annulus is measured and reported in millimeters. A value of TAPSE < 16 mm reflects RV systolic dysfunction [26,29]. The main limitations are that TAPSE is angle- and load-dependent [29] and that it measures the displacement of a single segment of the RV free wall. Furthermore, it does not account for the radial and antero-posterior contraction, and therefore, it does not reflect the global RV systolic function [30].

Load-dependency means that TAPSE will change with different loading conditions without actual changes in myocardial contractility. TAPSE decreases with increased pulmonary vascular resistance [31], but it also may be overestimated in patients with pulmonary hypertension and clockwise rotation of the heart due to LV compression [32]. TAPSE is also dependent on preload, being directly correlated with RV end-diastolic volume and overestimating RV function in patients with mild to moderate RV dilation [33].

Ghio et al. showed that TAPSE ≤ 14 mm is an independent predictor of death or emergency cardiac transplantation in patients with congestive HF [34]. Similarly, Venner et al. found TAPSE ≤ 15 mm to be an independent predictor of major adverse cardiovascular events (MACEs) in patients with idiopathic DCM [35]. Several other studies showed that TAPSE is an independent predictor of all-cause mortality in patients with HF [36,37,38]. The prognostic ability of TAPSE appears to be improved when combined with the echocardiographic estimation of pulmonary artery systolic pressure (PASP): a PASP ≥ 40 mm Hg combined with TAPSE **≤** 14 mm predict unfavorable outcomes in patients with HF, irrespective of its ischemic or non-ischemic etiology [9].

## 4. Tricuspid Lateral Annular Systolic Velocity (S’ Wave)

The systolic velocity of the tricuspid lateral annulus is measured in the apical four-chamber view by placing the tissue Doppler marker on the lateral tricuspid annulus [29,30]. Similar to TAPSE, it is an easy obtainable parameter, but it is angle-dependent, and it evaluates the longitudinal shortening and not the global systolic function of the RV [29,30]. An S’ wave value <9.5 cm/s reflects RV systolic dysfunction [26,29].

Studies found that decreased TDI systolic velocity of the tricuspid annulus is an independent predictor of either cardiac death [39,40] or cardiovascular death and rehospitalizations for HF [41] in patients with LV systolic dysfunction. Damy et al. showed that an S’ wave <9.5 cm/s is a strong independent predictor of outcomes in patients with LVEF <35%, with better prognostic value than FAC and TAPSE [42]. This could be explained by the lower variability of S’ wave measurement as compared to the other parameters. Another study found that both TDI systolic and diastolic velocities of the tricuspid annulus were independent predictors of survival and of event-free survival in HFrEF. In this study, patients with combined peak systolic velocity <10.8 cm/s and peak early diastolic velocity <8.9 cm/s had the worst prognosis [43].

## 5. Right Ventricular Myocardial Performance Index (RV MPI)

**Figure 1:**
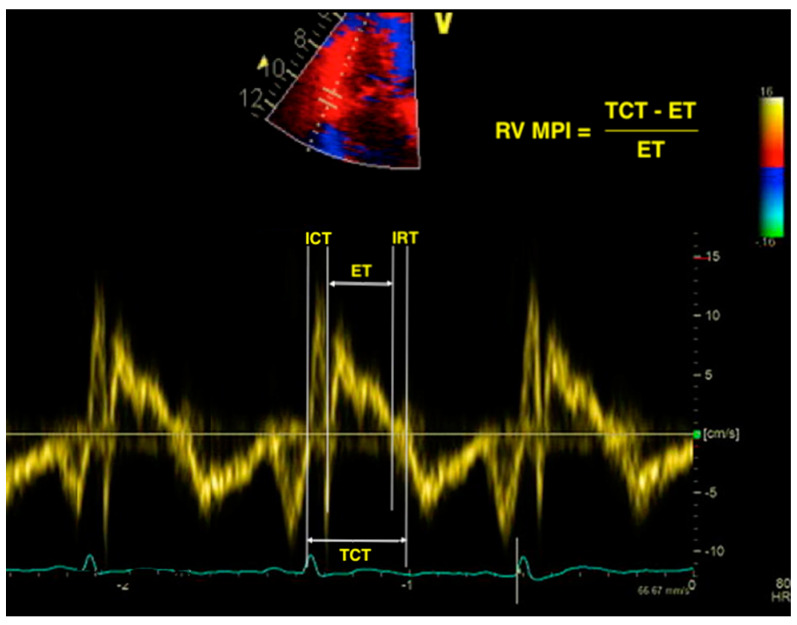
Calculation of right ventricular myocardial performance index (RV MPI) using the tissue Doppler imaging (TDI) method. RV—right ventricle; MPI—myocardial performance index; TDI—tissue Doppler imaging; ICT—isovolumic contraction time; ET—ejection time; IRT—isovolumic relaxation time; TCT—total contraction time.

The RV myocardial performance index, also known as the right Tei index, is a measure of both systolic and diastolic RV function. It is a unitless parameter, calculated by dividing the total isovolumic time (isovolumic contraction plus isovolumic relaxation) by the ejection time (ET) [30]. Systolic dysfunction prolongs the isovolumic contraction time (ICT) and shortens the ET, while prolonged isovolumic relaxation time (IRT) is encountered in both systolic and diastolic dysfunction. Therefore, impaired RV global function will lead to a high RV MPI. The parameter can be measured using either pulsed-wave Doppler or tissue Doppler (Figure 1). The proposed cutoff values for abnormal RV MPI are >0.43 using pulsed Doppler and >0.54 using tissue Doppler [26]. The advantage of RV MPI is that it bypasses the limitations of the complex RV geometry, as it is only derived from time intervals and makes use of no assumption of RV shape. However, it is unreliable in patients with elevated right atrial pressure, and irregular rhythms make MPI difficult to calculate [29,30].

The prognostic value of the pulsed-Doppler-derived RV MPI was assessed in a cohort of HFrEF patients, who were prospectively followed for 5 years for a combined endpoint of cardiac death and readmissions for HF. The authors found that an RV MPI > 0.38 was an independent predictor of adverse outcomes [44]. In a study by Field et al., each 0.1-unit increase in RV MPI was associated with a 16% increased risk of MACEs (defined as death, cardiac transplantation, or ventricular assist device placement) in patients with advanced HF referred for cardiac resynchronization therapy (CRT) [45]. To our knowledge, there are no studies to assess the prognostic role of TDI-derived RV MPI in HF. However, some authors suggest that TDI-derived MPI is superior to pulsed-Doppler-derived MPI because all the time intervals are measured during the same cardiac cycle [46].

## 6. Right Ventricular Fractional Area Change (RV FAC)

FAC is a 2D measure of RV systolic function obtained from the RV-focused apical four chamber view by manually tracing the endocardial border of the RV in end-diastole and end-systole. It is calculated as: (end-diastolic area − end-systolic area)/end-diastolic area × 100% [26]. The RV-focused view is acquired by laterally displacing and rotating the probe from the standard apical four-chamber view until the maximal RV basal and longitudinal diameters are obtained [47,48]. The measurements from the RV-focused view are more reproducible than those obtained from the apical four-chamber view [48]. RV FAC reflects both the longitudinal and radial shortening of the RV, but it neglects the contraction of the outflow tract [26,30]. It has shown good correlation with the RV ejection fraction (RVEF) determined by CMR [49], but it is load-dependent and potentially difficult to acquire in the case of poor endocardial definition [30,50]. An RV FAC < 35% reflects RV dysfunction [26,29].

Zornoff et al. found that RV FAC is an independent predictor of total mortality, cardiovascular mortality, and development of HF in patients with LV systolic dysfunction following a myocardial infarction (MI), with each 5% decrease in FAC being associated with a 16% increase in odds of cardiovascular mortality [51]. Similar findings were reported by Anavekar et al., who found RV FAC to be an independent predictor of all-cause mortality, cardiovascular death, sudden death, HF, and stroke in patients with MI and LV dysfunction [52].

A small retrospective study found that RV FAC < 26.7% is predictive of death or LV assist device implantation in patients with DCM, providing better prognostic value than TAPSE and S’ wave velocity [53]. Similar results were reported by Merlo et al., who found FAC < 35% to be an independent predictor of death or heart transplantation in patients with idiopathic DCM; moreover, RV FAC had stronger predictive value than other well-known prognostic factors such as LV dimensions and New York Heart Association (NYHA) functional class [54].

## 7. Right Ventricular Isovolumic Acceleration

**Figure 2:**
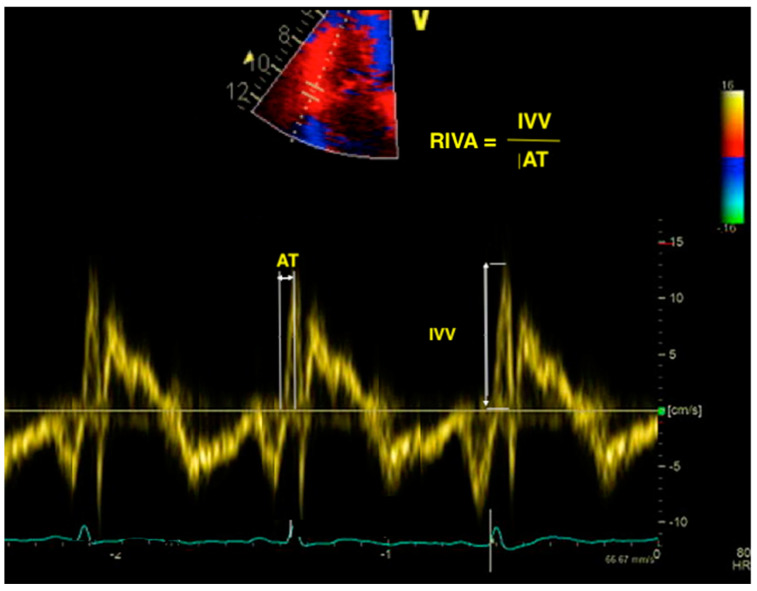
Calculation of RIVA using TDI. RIVA—right isovolumic acceleration; TDI—tissue Doppler imaging; IVV—isovolumic velocity; AT—acceleration time.

Myocardial acceleration during isovolumic contraction is usually obtained using TDI at the lateral tricuspid annulus in the apical four-chamber view. It is calculated as the peak myocardial velocity during isovolumic contraction divided by the time needed to reach this velocity (Figure 2). While it has the advantage of being relatively load-independent [30], it has a large confidence interval around the normal values [29]; hence, it is not recommended for routine use and no reference value for this parameter has been proposed by the latest guidelines [26]. Consequently, its prognostic utility has not been broadly studied. However, Sciatti et al. found RV isovolumic acceleration to be a better predictor for cardiac death and rehospitalization in patients with HF and reduced LVEF than traditional parameters of RV systolic function such as TAPSE, RV FAC, and S’ wave [55].

## 8. Right Ventricular Strain and Strain Rate Derived from Two-Dimensional Speckle-Tracking Echocardiography (2D STE)

**Figure 3:**
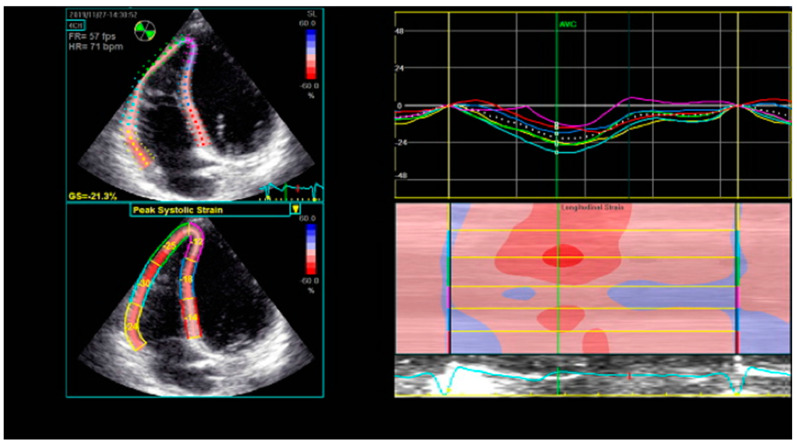
Six-segment model of longitudinal RV strain using STE. RV—right ventricle; STE—speckle-tracking echocardiography.

Speckle-tracking echocardiography is a non-invasive, innovative technique that analyzes the segmental myocardial deformation along different planes through the displacement of speckles [25]. Originally designed for the assessment of the LV, it is now also being applied for the analysis of RV deformation. Strain represents the percentage change in length of a myocardial segment, while strain rate represents the rate of deformation over time [56]. Both strain and strain rate are indices of myocardial contractility [57]. The RV longitudinal strain and strain rate may be measured in the apical RV-focused four-chamber view, using the software dedicated for the LV assessment. The RV free wall and the interventricular septum (IVS) are each divided into three segments (basal, medial, and apical), providing a six-segment model (Figure 3). The global longitudinal strain of the RV is calculated as the average of the six segmental values, while the longitudinal strain of the RV free wall (RVFW) is calculated as the average of the three segmental values of the free wall [58]. The latter is considered to be more specific for the RV [25], since the motion of the IVS contributes to both RV and LV function.

STE assesses the deformation of myocardial speckles in two dimensions along the myocardial wall direction, thus being less confounded by the motion of the heart [59] and relatively angle-independent when compared to TDI-derived parameters [60]. The advantages of 2D-STE-derived strain are the angle independence, the relative load independence, the strong correlation with RVEF measured by CMR [61], and the ability of detecting subtle myocardial abnormalities, which cannot be identified using conventional parameters [62,63]. One study showed that RVFW strain had a good correlation with the extent of myocardial fibrosis detected on CMR [64]. However, there is no uniformity among software and no reference range agreement between vendors. Other drawbacks are that strain assessment is dependent on good image quality, it is influenced by artifacts, and it neglects the contribution of the RV outflow tract (RVOT) to the global RV performance [26]. For the longitudinal strain of the RV free wall, a value > −20% is considered abnormal [26].

Martin et al. analyzed which of the RV strain parameters was a better predictor of hospitalizations for HF in patients with left heart disease. They showed that the RV global longitudinal strain independently predicts readmissions, providing additional prognostic information to that obtained by TAPSE [65]. Similar findings were reported by Motoki et al., who found global RV strain to be an independent predictor of long-term adverse outcomes in patients with LVEF < 35%, while RVFW strain was not. In their study, a global RV strain > −14.8% independently predicted the primary endpoint of death, cardiac transplantation, or hospitalization for HF at 5 year follow-up [66]. This is contrary to the results of another study, which found that RVFW strain was a better outcome predictor than global RV strain in HFrEF, as it independently predicted total mortality and readmissions for HF [67]. Another prospective study showed that an RVFW strain > −21% in patients with HF is an independent predictor for a composite endpoint of death, acute HF, emergency transplantation, or left ventricular assist device (LVAD) implantation at 1 year [68].

Carluccio et al. proved the superiority of RV strain over TAPSE, by following 200 patients with HFrEF but preserved TAPSE (>16 mm) for a composite endpoint of death and HF rehospitalization. The authors found that the RVFW longitudinal strain was an independent predictor of adverse outcome, with a cutoff value for endpoint prediction of −15.3% [69]. In a recent study by Seo et al., 143 patients with DCM were prospectively followed for long-term unfavorable events (defined as all-cause death, cardiac death, aborted sudden cardiac death, and HF hospitalization), for a median period of 40 months. The RVFW longitudinal strain was the only independent predictor of the primary outcome, with an optimal cutoff value for event prediction of −16.5% [70].

Several studies discovered independent prognostic roles for both global RV strain and RVFW strain in HFrEF. Cameli et al. found that in patients with advanced systolic HF referred for cardiac transplantation, both global and free-wall RV strain are independent predictors of an adverse outcomes (defined as cardiac death, heart transplantation, LVAD placement, intra-aortic balloon pump implantation, or acute HF), with stronger predictive power than other conventional parameters, including parameters of LV function [71]. Another study reported that both global RV strain and RVFW strain are independent predictors of all-cause mortality in patients with HF and LVEF < 45% [72]. A recent study by Houard et al. evaluated the prognostic value of 2D RV strain for survival prediction and compared it with conventional echocardiographic parameters and CMR in 266 patients with HF and reduced EF. The authors found out that both global RV strain and RVFW strain were independent predictors for overall mortality and cardiovascular mortality; moreover, the predictive power of RV strain was higher than that of FAC, TAPSE, CMR-derived RVEF, and CMR-derived RV strain [73].

## 9. Three-Dimensional Right Ventricular Ejection Fraction (3D RVEF)

**Figure 4:**
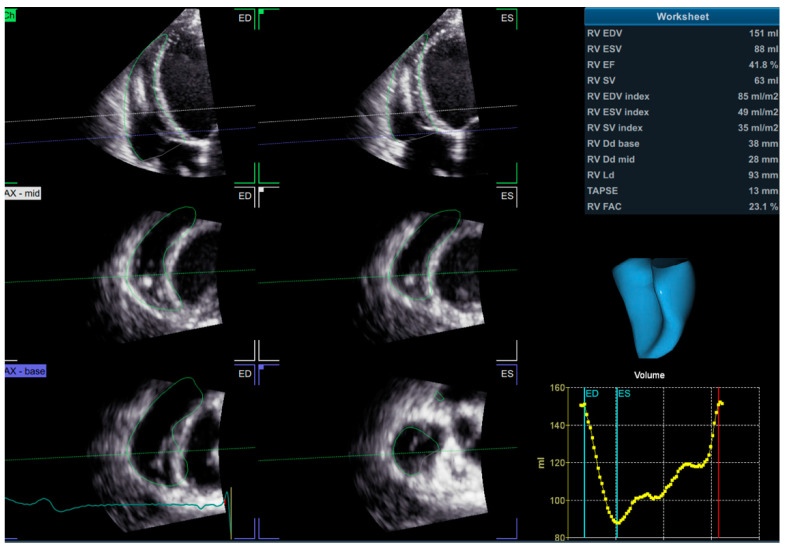
3D assessment of RV volumes and ejection fraction using dedicated software. 3D—three dimensional; RV—right ventricle.

3D echocardiography overcomes the geometric assumptions used in 2D echocardiography. As such, it is particularly useful for the evaluation of the RV, which—due to its complex anatomy—cannot be comprehensively assessed with 2D measurements only. 3D echocardiography integrates both the longitudinal and radial components of RV contraction [30] and, unlike 2D echocardiography, allows the assessment of antero-posterior shortening as well. The images are acquired with a 3D probe from the apical RV-focused view, usually using a full-volume data set and a multi-beat acquisition. The acquired image must include the entire RV volume, from the tricuspid valve to the pulmonary valve, with good temporal and spatial resolution. The data set is subsequently analyzed with dedicated software (Figure 4), by tracing the endocardial surface of the RV, which allows the reconstruction of the RV geometry and the calculation of RV volumes and EF. The 3D RV volumes and EF have been widely validated against the gold standard represented by CMR [74,75,76]. The main limitations of 3D RVEF are load dependency, challenges in correctly tracing the endocardial border, image quality, “stitching” artefacts in the case of arrhythmias, time consumption, and limited availability [30]. A 3D RVEF < 45% is considered abnormal [26].

In a population-based cohort study that enrolled 1004 elderly people, Nochioka et al. used 2D and 3D echocardiography to analyze the prevalence and prognostic role of RV dysfunction in HF. Among patients with no HF at baseline, 3D RVEF proved to be an independent predictor of death or incident HF: each 5% decrease in 3D RVEF was associated with a 20% increase in the hazard of death or hospitalization for HF, independent of LVEF [77].

Magunia et al. found that 3D RVEF is an independent predictor of post-operative RV failure in LVAD recipients [78], which is a well-known, common cause of mortality after LVAD implantation [79]. In a recent study, the long-term prognostic value of 3D RVEF was evaluated in 446 patients with various cardiovascular diseases, who were followed during 4.1 years for a primary endpoint of cardiac death and a secondary composite endpoint of cardiac death, ventricular fibrillation, nonfatal myocardial infarction, and hospitalization for HF exacerbation. At the end of the follow-up period, 3D RVEF was found to be an independent predictor of both cardiac death and of the secondary endpoint of MACEs [80].

A recent retrospective study of Surkova et al. evaluated the relative importance of different combinations of reduced and preserved 3D LVEF and 3D RVEF in predicting mortality in patients with different cardiac diseases. Reduced 3D RVEF, but not LVEF, was a strong and independent predictor of both all-cause mortality and cardiovascular mortality [81]. Moreover, 3D RVEF was superior to conventional echocardiographic parameters of RV performance to predict total mortality. The group of patients with reduced LVEF and reduced RVEF had the highest mortality in the study; interestingly, patients with reduced LVEF and preserved RVEF had significantly better survival than patients with reduced RVEF and preserved LVEF [81]. The results of this study draw attention to the potential role of therapies targeting RV dysfunction to improve clinical outcome.

## 10. Three-Dimensional Speckle-Tracking Echocardiography (3D STE)

3D STE is a novel imaging technique that evaluates myocardial deformation using 3D full-volume data sets. It is currently limited to research and not available for routine use, but it appears to be a promising tool for the assessment of the complex RV myocardial motion [82]. A study by Field et al. showed favorable results concerning the ability of 3D STE to detect subclinical biventricular dysfunction after anthracycline chemotherapy [45]. Another study found that 3D strain of the RVFW is a predictor of mortality in LVAD recipients [78]. Smith et al. evaluated the utility of 3D STE for RV assessment in a cohort of patients with pulmonary hypertension of different etiologies (including left heart disease) and found out that RV area-strain derived from 3D STE correlated well with RVEF and was an independent predictor of mortality [83].

## 11. Other Parameters of Right Ventricular Function

The interaction between the RV and the pulmonary circulation unit is reflected in the RV–pulmonary artery coupling (RVPAC), which is usually assessed with right heart catheterization. This parameter reflects the adaptation of the RV to afterload, and it is calculated from invasive pressure–volume loops as the ratio of RV end-systolic elastance to pulmonary artery elastance [84]. Several echocardiographic studies used the ratio between TAPSE and PASP as a non-invasive surrogate for the RVPAC, as this ratio, which reflects the interaction between the shortening of the RV fibers and the force generated by the RV, showed good correlation with invasively measured RVPAC [85]. The TAPSE/PASP ratio was found to be an independent predictor of cardiac mortality [86] and of major events (cardiac death, heart transplant, or LVAD implant) [87] in patients with HF. In a recent study, Ghio et al. enrolled 1663 patients with HF (1123 with reduced LVEF, 156 with mid-range LVEF, 384 with preserved LVEF) and showed that TAPSE/PASP is a powerful, independent predictor of all-cause mortality in all HF patients, regardless of the extent of LV dysfunction [88]. Similar results were found by Bosch et al., in a study that assessed the contribution of RV dysfunction in HFrEF versus HF with preserved EF (HFpEF); they showed that TAPSE/PASP ratio was an independent predictor of all-cause death and HF hospitalization, with no difference between HFrEF and HFpEF and regardless of LVEF [89].

As innovative echocardiographic techniques become part of the comprehensive assessment of RV performance, some researchers used 2D RV longitudinal strain or 3D RVEF for the non-invasive estimation of RVPAC, which was calculated as either RV strain/PASP ratio [89,90] or as 3D RVEF/PASP ratio [77]. One recent study found that the ratio between RVFW strain and PASP independently predicted a composite endpoint of all-cause death and rehospitalizations in patients with HF [89]. Similar results were found by Iacoviello et al., who showed that both RVFW strain/PASP ratio and global RV strain/PASP ratio are independent predictors for all-cause mortality in patients with HF and LVEF < 45% [90]. In another study, RVPAC was estimated non-invasively using the ratio between 3D RVEF and PASP; the authors found that each 0.5 unit decrease in RVEF/PASP ratio was associated with a 65% increase in the hazard of death or hospitalization for HF [77].

Fractional shortening of the RVOT (RVOT-FS) is an index of RV performance that is obtained using M-mode echocardiography in the parasternal short axis window at the level of the aortic root. It is calculated as the percentage change in RVOT diameter at end-systole compared to end-diastole [91]. Several studies showed a good correlation between RVOT-FS and other indices of RV systolic performance [92,93]. Yamaguchi et al. showed that RVOT-FS is an independent predictor of MACE (defined as cardiac death, heart transplantation, or hospitalization for HF) in a cohort of patients with LVEF < 40%, with a higher rate of adverse outcome in patients with RVOT-FS < 20% [94].

The above-mentioned studies evaluating the prognostic role of RV dysfunction in HF are summarized in Table 1.

## 12. Artificial Intelligence Algorithms

Artificial intelligence (AI) techniques, such as machine learning (ML) and deep learning, can improve the diagnostic accuracy of echocardiography, by providing fully automated image analysis and thus potentially reducing human error [95]. So far, only one study evaluated an ML-based software for 3D echocardiographic quantification of the RV. The algorithm provided accurate and reproducible measurements for RV volumes and function, showing good correlation with CMR [96]. Further research is still needed in order to refine and validate such algorithms and to establish their utility in routine clinical practice. However, AI-based approaches hold great promise to improve the echocardiographic quantification of the RV.

## 13. Conclusions

The RV plays a crucial role in various clinical settings. RV dysfunction is a strong independent predictor of mortality and adverse outcomes not only in diseases of the right heart or pulmonary vascular bed but also in diseases primarily involving the LV. In the particular setting of DCM, RV FAC appears to be a better outcome predictor than other conventional RV parameters, while RVFW strain has a higher prognostic value than global RV strain. There is no perfect single parameter that comprehensively evaluates RV performance. Integrating novel techniques in the RV echocardiographic assessment allows a better evaluation and an enhanced risk stratification for patients with HF, thus improving therapeutic strategies and potentially leading to an improved outcome.

## Figures and Tables

**Table 1:** Selection of studies demonstrating an independent prognostic role of RV functional parameters in patients with HF.

Study	Publication Year	Number of Patients	Study Type	Parameter	Proposed Cutoff
Dokainish et al. [41]	2007	107	Prospective	S’ wave	9 cm/s
Damy et al. [42]	2009	136	Prospective	S’ wave	9.5 cm/s
De Groote et al. [40]	2012	527	Prospective	S’ wave	9.7 cm/s
Vizzardi et al. [44]	2012	95	Prospective	RV MPI	0.38
Dini et al. [37]	2012	373	Prospective	TAPSE	14 mm
Damy et al. [38]	2012	1547	Prospective	TAPSE	15.9 mm
Guazzi et al. [86]	2013	293	Prospective	TAPSE/PASP	0.36
Yamaguchi et al. [94]	2013	81	Prospective	RVOT-FS	20%
Motoki et al. [66]	2014	171	Retrospective	Global RV strain	−14.8%
Sciatti et al. [55]	2015	60	Prospective	RIVA	1.5 m/s^2^
Garcia-Martin et al. [65]	2016	103	Prospective	Global RV strain	−17.3%
Iacoviello et al. [72]	2016	332	Prospective	Global RV strain, RVFW strain	−14%, −20.6%
Venner et al. [35]	2016	136	Retrospective	TAPSE	15 mm
Merlo et al. [54]	2016	512	Retrospective	FAC	35%
Kawata et al. [53]	2017	68	Retrospective	FAC	26.7%
Ghio et al. [88]	2017	1663	Retrospective	TAPSE/PASP	0.36
Bosch et al. [89]	2017	438	Prospective	TAPSE/PASP, global RV strain/PASP	0.48, −0.56
Iacoviello et al. [90]	2017	315	Prospective	RV strain/PASP, RVFW strain/PASP	−0.36, −0.66
Nagata et al. [80]	2017	446	Prospective	3D RVEF	35% for cardiac death, 41% for MACE
Seo et al. [70]	2019	143	Prospective	RVFW strain	−16.5%
Houard et al. [73]	2019	266	Prospective	Global RV strain	−19%
Carluccio et al. [67]	2019	288	Prospective	Global RV strain, RVFW strain	−14.6%, −15.3%
Surkova et al. [81]	2019	394	Prospective	3D RVEF	45%

Abbreviations: S’ wave—systolic velocity of the tricuspid lateral annulus; RV—right ventricular; MPI—myocardial performance index; TAPSE—tricuspid annular plane systolic excursion; PASP—pulmonary artery systolic pressure; RVOT-FS—right ventricular outflow tract fractional shortening; RIVA—right ventricular isovolumic acceleration time; RVFW—right ventricular free wall; FAC—fractional area change; 3D—three dimensional; RVEF—right ventricular ejection fraction.
